# An Indoor Continuous Positioning Algorithm on the Move by Fusing Sensors and Wi-Fi on Smartphones

**DOI:** 10.3390/s151229850

**Published:** 2015-12-11

**Authors:** Huaiyu Li, Xiuwan Chen, Guifei Jing, Yuan Wang, Yanfeng Cao, Fei Li, Xinlong Zhang, Han Xiao

**Affiliations:** 1 Institute of Remote Sensing and GIS, Peking University, No. 5 Yiheyuan Road, Haidian District, Beijing 100871, China; xwchen@pku.edu.cn (X.C.); pkuwangy@pku.edu.cn (Y.W.); caoyanfeng@cate.casc (Y.C.); gisor.lee@pku.edu.cn (F.L.); 1501110619@pku.edu.cn (X.Z.); hxiao@pku.edu.cn (H.X.); 2 National Remote Sensing Center of China, No. 8A, Liulinguan Nanli, Haidian District, Beijing 100036, China; jinggf@most.cn; 3 Beijing Aerospace Unmanned Vehicles System Engineering Research Institute, No. 1 Fengyingdong Road, Haidian District, Beijing 100094, China

**Keywords:** indoor positioning, Wi-Fi, PDR, multi-sensor fusion, TCPF

## Abstract

Wi-Fi indoor positioning algorithms experience large positioning error and low stability when continuously positioning terminals that are on the move. This paper proposes a novel indoor continuous positioning algorithm that is on the move, fusing sensors and Wi-Fi on smartphones. The main innovative points include an improved Wi-Fi positioning algorithm and a novel positioning fusion algorithm named the *Trust Chain Positioning Fusion* (*TCPF*) algorithm. The improved Wi-Fi positioning algorithm was designed based on the properties of Wi-Fi signals on the move, which are found in a novel “quasi-dynamic” Wi-Fi signal experiment. The *TCPF* algorithm is proposed to realize the “process-level” fusion of Wi-Fi and Pedestrians Dead Reckoning (PDR) positioning, including three parts: trusted point determination, trust state and positioning fusion algorithm. An experiment is carried out for verification in a typical indoor environment, and the average positioning error on the move is 1.36 m, a decrease of 28.8% compared to an existing algorithm. The results show that the proposed algorithm can effectively reduce the influence caused by the unstable Wi-Fi signals, and improve the accuracy and stability of indoor continuous positioning on the move.

## 1. Introduction

Indoor positioning technology based on smartphones has many application scenarios since people stay inside buildings more than 80% of their daily life [1,2]. Due to the advantages of low cost, high accuracy, and wide popularization, Wi-Fi indoor positioning has become one of the mainstream indoor positioning technologies. After the RADAR system [3,4] firstly put forward the Wi-Fi indoor fingerprint positioning scheme, many indoor positioning algorithms based on its framework were proposed, such as probability positioning algorithms [5,6,7] based on the Bayesian estimation, and machine learning algorithms [8,9,10] including Support Vector Machine (SVM) and neural network. As a result, The indoor positioning accuracy has been further improved, achieving about, on average, a 3 m positioning accuracy, which approximates to the size of a typical office. Current Wi-Fi positioning algorithms impose the impractical prerequisite that users and terminals remain at a fixed location during the positioning process [11]. However, the application scenarios of indoor positioning are most focused on smartphones, which leads to many positioning problems on the move, such as large positioning error, positioning jumps and accuracy reduction [12,13]. There are few researches focusing on the improvement of Wi-Fi positioning algorithms on the move.

Another direct method to improve positioning accuracy is fusing two or more complementary technologies. With the improvement of integration and the power consumption reduction of multi-sensors in recent years, more and more sensors are integrated in smartphones. Algorithms for fusing sensors and Wi-Fi have become a research hotspot [14]. Based on built-in sensors, the PDR positioning can calculate the relative displacement to realize the indoor positioning on smartphones. As a relative positioning method, the PDR positioning has a high accuracy over a short distance, and it needs a reference point as a starting point. However, the most serious problem is that the error will be accumulated over time. On the contrary, the Wi-Fi positioning belongs to the absolute positioning method, which will not accumulate the positioning error. These two positioning algorithms are complementary to each other so that the fusion can prominently improve the performance of indoor positioning.

The current fusion algorithms mainly include algorithms based on Particle Filter [15,16], algorithms based on Kalman Filter [17,18,19,20,21,22,23,24], the Cross-Assistive algorithm [12,13,25], and so on. The algorithm based on Particle Filter has an intuitive process, but the large amount of computation is not suitable for a handheld device [15,17,18]. The algorithm based on Kalman Filter has a good real-time performance, but the fusion is on the “result-level” so that positioning results will be easily skewed by Wi-Fi signal interferences under volatile conditions. The Cross-Assistive algorithm is currently proposed to achieve deep fusion in the Wi-Fi/PDR positioning process. However, the algorithm is not stable, and is prone to fall into error cycles.

Overall, we put forward a novel indoor continuous positioning algorithm fusing built-in sensors and Wi-Fi on smartphones. Compared to traditional algorithms, it has two innovative points. The first one is an improved Wi-Fi positioning algorithm, and the other is a new positioning fusion algorithm named *TCPF* algorithm. Through these two improvements, the proposed algorithm can optimize indoor positioning performance of targets on the move.

The following sections are arranged as follows. The previous related researches will be reviewed in Section 2. A novel “quasi-dynamic” Wi-Fi signal experiment is conducted to analyze the properties of Wi-Fi signals on the move, and an improved Wi-Fi positioning algorithm will be proposed in Section 3. The novel positioning fusion algorithm, including trusted point determination, trust state and positioning fusion algorithm, is introduced after the overall framework of the indoor continuous positioning algorithm is outlined in Section 4. Another field indoor experiment was conducted to verify the proposed algorithm, and the results are analyzed in three parts in Section 5. Conclusions and the future research direction are summarized in Section 6.

## 2. Related Work

Researches on the Wi-Fi positioning field focus on positioning algorithm improvement [10], signal analysis [26,27], fingerprint database construction [28], and so on. Their fundamental purpose is to improve Wi-Fi positioning performance, which is greatly affected by the effective restoration of actual long-time signals from the short-time signals gathered by terminals. Through analyzing the statistical properties of a large number of Wi-Fi signals, many researchers tried to analyze and explain the factors affecting indoor positioning accuracy and stability.

Kamol Kaemarungsi *et al.* analyzed the signal distribution, mean, standard deviation, deviation and stability of Wi-Fi Received Signal Strength Indicator (RSSI) from the perspective of indoor positioning systems [26]. Jiayou Luo *et al.* analyzed the properties of Wi-Fi RSSI distribution and differences among smartphones with the purpose to improve the Wi-Fi indoor positioning accuracy [27]. However, current Wi-Fi positioning algorithms are mostly based on the signal distributions of terminals at stationary state; there is a lack of research on the properties of Wi-Fi signals on the move and corresponding positioning algorithm improvements.

Fusing with inertial positioning is another focus. The existing fusion algorithms mainly include the algorithm based on Particle Filter [15,16], the algorithm based on Kalman Filter [17,18,19,20,21,22,23,24], and the Cross-Assistive algorithm [12,13,25]. The advantages and disadvantages of the three algorithms are shown as Table 1.

**Table 1:** A comparison of existing fusion algorithms.

	Advantages	Disadvantages
Based on Particle Filter	Clear and intuitive process Integrated with map matching	Large computations
Based on Kalman Filter	Good real-time performance Simple but effective	Fusion based on positioning result level Susceptible to Wi-Fi interference
Cross-Assistive Approach	Wi-Fi positioning filtered by PDR Positioning update by accurate result	There is inevitably some error cycles Susceptible to Wi-Fi interference

The Wi-Fi/INS fusion indoor positioning algorithm based on Particle Filter was successively proposed by Frederic Evennou *et al.* [15] and Hui Wang *et al.* [16]. A large number of particles are used to fit the discrete probability density function of the target. The spread of the particles is controlled by the acceleration at a random process, and map information is integrated to filter out the unreasonable movement, thus final position estimation is obtained. The algorithm has an intuitive and effective fusion process, however, it is time consuming since every step needs certain operations for each particle, and the number of particles can reach thousands, or even more. Overall, the algorithm based on Particle Filter is not suitable for smartphones whose computing power and resources are limited [15,17,18].

Kalman Filter algorithm is the core of the GPS/INS integrated navigation algorithm, which can also be specifically applied to the Wi-Fi/PDR fusion positioning. Zhenghua Chen *et al.* studied the Wi-Fi/PDR fusion positioning algorithm based on Kalman Filter [17]. Further, Zhi-An Deng *et al.* [18] and Veerachai Malyavej *et al.* [22] respectively studied the improved forms of Kalman Filter, Extended Kalman Filter (EKF), and Unscented Kalman Filter (UKF). Simo Ali-Loytty *et al.* [24] proposed an Fingerprint Kalman Filter (FKF) on the basis of the EKF and UKF. The algorithm based on Kalman Filter demonstrated a good real-time performance, but the fusion process is based on the level of positioning results. When Wi-Fi signal fluctuates under the condition of severe interference, hops will appear in the Wi-Fi positioning results, and deviations will be fused into the final location estimation so that the positioning stability needs further improvement.

The Cross-Assistive positioning algorithm is proposed for Wi-Fi/PDR fusion in the latest three years. K. Miyazaki *et al.* [12] proposed this algorithm to limit the fingerprint search scope based on PDR estimation and to use accurate Wi-Fi positioning results to correct the PDR positioning in order to achieve a finer fusion process. Chang Qiang *et al.* [13] further put forward an Error Distance to replace Euclidean Distance. However, there are some defects, such as the error cycle. Positioning fusion is triggered by the “accurate” Wi-Fi positioning results to fix the PDR positioning cumulated error, but Wi-Fi positioning results may experience a large deviation over a period of time due to large disturbances. In this case, fusion positioning results cannot be corrected, error is accumulated in the PDR positioning, giving no “accurate” positioning results, and finally the positioning deviates from the track. Another problem is using the sector area established by the PDR positioning and the latest location to determine whether positioning is accurate, because there is no bias in Wi-Fi positioning error so step length would be overestimated.

Other fusion algorithms include the sequential Monte Carlo filter, which was developed by the joint research between the University of California, Santa Barbara (UCSB) and Massachusetts Institute of Technology (MIT), fusing built-in inertial sensors with Wi-Fi to realize indoor navigation and positioning [29].

## 3. An Improved Wi-Fi Positioning Algorithm

The Wi-Fi fingerprint positioning algorithm usually works in two phases: an offline training phase and an online positioning phase. During the offline phase, the system tabulates the signal strength received from the access points (APs) at selected locations (which are called fingerprint points) in the area of interest, resulting in a so-called radio map. During the online positioning phase, the system uses the signal strength samples received from the APs to “search” the radio map and estimate the user location. Classic Wi-Fi positioning algorithm is the Weighted K Nearest Neighbor (WKNN [9]) algorithm, which is the improved version of the RADAR [3] system’s Nearest Neighbor (NN) algorithm. The estimated location is the weighted sum of the locations of the K fingerprint points, which have the minimum signal space Euclidean distance in the fingerprint database. The signal space distance can be expressed as Equation (1)

(1) Lqi=(∑j=1n(Sj−Sij)q)1q

where n is the dimension of the Wi-Fi signal in the fingerprint database. $S_{j}$ is the RSSI referred to the jth AP sampled during the online phase, and $S_{ij}$ is that during offline phase at the ith fingerprint points. When q is set 1, $L_{qi}$ is the Manhattan distance. When q is set 2, $L_{qi}$ is the Euclidean distance, which is adopted in this paper. Compared to the NN algorithm, the WKNN algorithm improves the positioning performance by fusing the K nearest fingerprint points (ordered according to $L_{i}$), as shown in Equations (2) and (3).

(2) $$w_{i}=\frac{\frac{1}{L_{i}}}{\sum_{i=0}^{K}\frac{1}{L_{i}}}$$

(3) $$\left(\hat{x}，\hat{y}\right)=\overset{K}{\underset{i=1}{\sum }}w_{i}\left(x_{i},y_{i}\right)$$

where $L_{i}$ is the signal space Euclidean distance referred to the ith fingerprint point. K is the number of selected fingerprint points, and $w_{i}$ is the weight of the ith one.

The stationary positioning accuracy is about 3 m [9], by using the classic WKNN algorithm in an ideal positioning environment without electromagnetic interference and crowds. However, when the user is on the move, or influenced by small scale fluctuations [7], there will be problems, such as a big positioning error and poor stability. The properties of Wi-Fi signals, especially at moving state, need to be deeply analyzed in order to improve the Wi-Fi positioning performance.

### 3.1. Properties of Wi-Fi Signals on the Move

Wi-Fi follows the 802.11× series standard. The measured value of the RSSI is the received instantaneous radio energy, which is the baseband IQ power integration in 10^4^ μs after a reverse channel baseband filter. The calculation of RSSI can be expressed as Equation (4), where the unit of RSSI is dBm, and $P_{0}$ is set as 1 mW.

(4) $$RSSI=10\log(\frac{\text{P}}{P_{0}})$$

However, it is insufficient to describe the properties of Wi-Fi signals on the move. We propose two new parameters, “refresh rate” and “loss rate”. Two adjacent Wi-Fi RSSI measurements have a minimum time interval, which is corresponding to the fastest refresh frequency. When the connection is good, the Wi-Fi signal refresh frequency can maintain the fastest rate. The rate between the current Wi-Fi signal refresh frequency and the fastest one is calculated as the “refresh rate”. The “loss rate” is the mean probability of the terminal losing the Wi-Fi signal in the continuous measurements. Through continuously measuring the Wi-Fi RSSI, the number of null values divides the total number of measurements to get the “loss rate”. These two parameters are used to quantify the instability of Wi-Fi signals. The smaller the “refresh rate” is, or the larger the “loss rate” is, the less reliable the current signal is. This paper focuses on the “refresh rate”, and the “loss rate” can be deduced in a similar way.

According to the measurement principle of RSSI, the RSSI measurement has a power threshold $P_{r}$. When the received signal power is stronger than the $P_{r}$, the signal can be accurately captured. Otherwise, the signal cannot be easily captured, resulting in none refreshing RSSI. It is concluded that the “refresh rate” should be inversely proportional to the signal strength, which is exponentially related to the RSSI. Overall, the relationship model between the “refresh rate” (η) and the RSSI is shown in Equation (5), where the parameters $\eta_{0},a,b,c$ are all real numbers greater than zero. The model is named Refresh Rate model of Wi-Fi signals on the move (RR).

(5) $$\eta \text{=}\eta_{0}+\frac{a}{1+{\left(\frac{P_{r}}{P}\right)}^{b}}=\eta_{0}+\frac{a}{1+e^{\left(-\frac{RSSI-RSSI_{r}}{c}\right)}}$$

**Figure 1:**
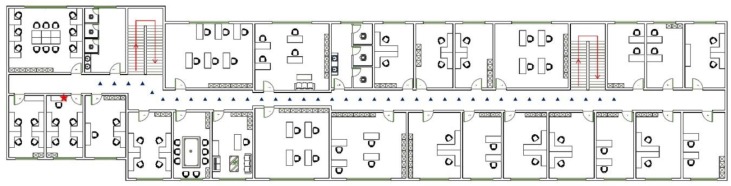
“Quasi-dynamic” Wi-Fi signal experimental indoor layout.

We set up a “quasi-dynamic” Wi-Fi signal experiment (called Experiment 1) at Remote Sensing Building in Peking University. The indoor layout of experiment area is shown in Figure 1. One AP is put in Room 404 with a marker “★” and the “quasi-dynamic” Wi-Fi signal measurement is taken at the place with the marker “▲”. Measurements are made 20 times at each point with 0.5 s intervals. Adjacent point interval is 1 m and the total number of points is 38. All points are separated by a door from the AP, which is representative of the most typical indoor environment. Different from the terminal statically measuring Wi-Fi signals in the traditional experiment, the “quasi-dynamic” measurement means that the terminal keeps moving to measure Wi-Fi signals at each measurement point where the user is. The terminal was held at the palm, swinging with the hand. The user is standing stationary at the fixed point without moving the feet and the body.

**Figure 2:**
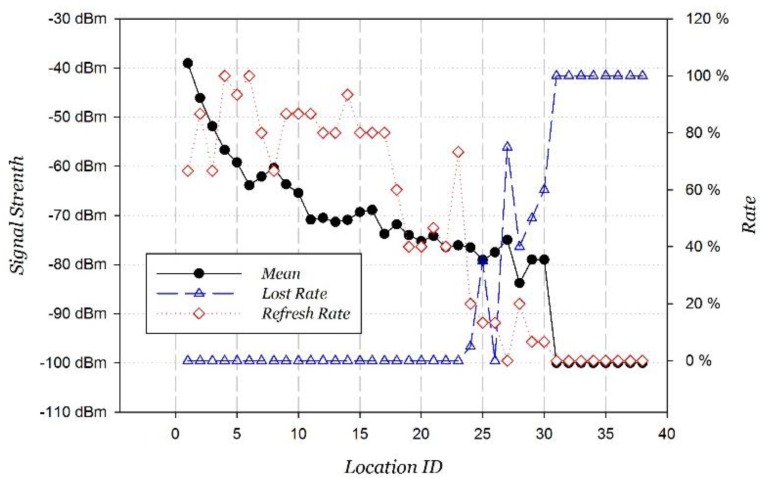
Wi-Fi variation on the move.

The variations of the average RSSI, “refresh rate” and “loss rate” of Wi-Fi signals as the distance from the AP increases are shown in Figure 2. Two interesting discoveries from the results are listed below:

1. When the Wi-Fi signal quality is good (RSSI is stronger than −70 dBm), the refresh frequency is high, being maintained within 1 s (minimum is 0.667), and the “loss rate” is 0 without packet loss. In other words, at this time, the Wi-Fi signal is reliable.
2. When the Wi-Fi signal quality is bad (RSSI is weaker than −80 dBm), the “refresh rate” quickly decreases and the “loss rate” quickly increases, nearly to zero, as the RSSI decreases. In other words, at this time, the Wi-Fi signal is unreliable.

**Figure 3:**
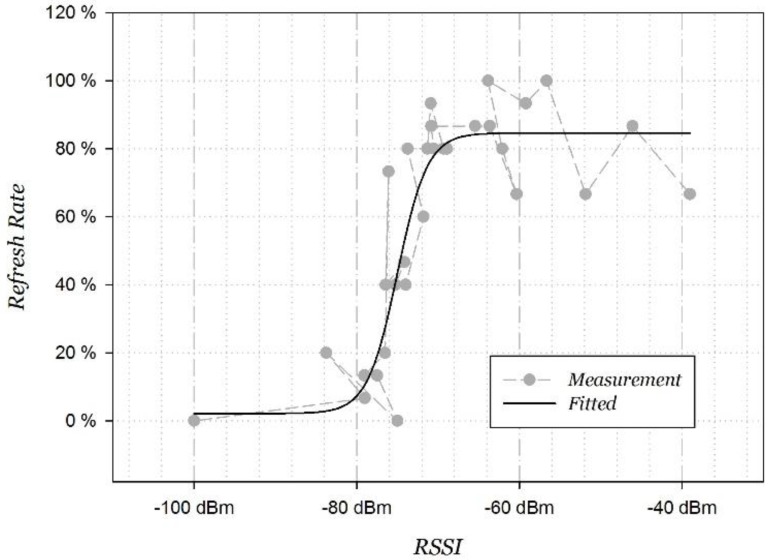
Curve fitting refresh rate function.

The fitted curve of RR model according to Equation (5) is shown in Figure 3, where $\eta_{0}=2.1630$, a=82.4917, $RSSI_{r}=-75.0075$, c=1.8464, and the fitting correlation coefficient is 0.9285. There is an obvious inflection point around −75 dBm in the fitted curve. The “refresh rate” rapidly decreases as the RSSI decreases. It happens to conform with the design of the Wi-Fi signal; the signal is defined as weak when its RSSI is between −75 dBm and −85 dBm.

The above statistical distribution partly explains the deterioration of Wi-Fi positioning performance at moving state. There is no different consideration given for stationary or moving states in traditional Wi-Fi positioning algorithms. When the terminal is online positioning at stationary state, Wi-Fi signals can be sampled many times and be well estimated through a mean filter with a sliding window so that weak signals can still be accurately captured, and they are well matched with the offline data. However, the “refresh rate” of the weak signal is low, and the “loss rate” is high when the terminal is moving with a continuous positioning. At this time, due to the cache mechanism of the base hardware in sampling signals, the RSSI measurement deviates from the actual value. It leads to the mismatch with the offline data so that increases the probability of unstable positioning and large positioning errors.

### 3.2. Algorithm Improvement

According to the properties of Wi-Fi signals on the move, we put forward two methods including dynamically adjusting the RSSI threshold and AP matching.

#### 3.2.1. Dynamic Adjustment of RSSI Threshold

According to the fitted distribution of the “refresh rate”, Wi-Fi signals on the move vary dramatically near −75 dBm. We put forward the method to dynamically adjust the RSSI threshold based on the user’s motion state. By analyzing the mean and variance of the horizontal acceleration (the component perpendicular to the gravity acceleration), and variance of the resultant acceleration, the static state can be accurately identified when all these three parameters are less than a certain threshold, respectively [30]. The simplest way is to set the threshold as −85 dBm at stationary state, and the corresponding −75 dBm at moving state, respectively. Of course, the threshold can also be dynamically adjusted according to other motion states.

#### 3.2.2. AP Match

We can see from Figure 2 that the RSSI decreases quickly near the AP (within 10 m). The signal then decays slowly until reaching a threshold, where the “refresh rate” decreases, the “loss rate” increases rapidly, and the signal gradually disappears. Considering that adjacent APs usually have a certain distance in the real set, the strong RSSI of Wi-Fi signal has a significant clustering feature.

Here, we put forward the AP matching method. Considering the relative stability of the strong signal in dynamic positioning and the overlapping area between the adjacent APs, offline fingerprints with two strongest APs containing the strongest AP of the online signal are selected to calculate the Wi-Fi positioning, as shown in Equation (6).

(6) $${\text{AP}}_{\max,\mathrm{online}}\in \left\{AP_{top2,offline}\right\}$$

The clustering is done in the offline training phase to build up the associated fingerprint database. The codes of two strongest APs are extracted from fingerprint points as their indices. When the target is at stationary or moving state, it would be easy to use the AP matching method to find the candidate fingerprint points instead of searching through the database. Only matched fingerprint points can be selected to calculate the Wi-Fi positioning so that large positioning errors could be avoided. The AP matching algorithm can effectively reduce the amount of online data that need to be considered and calculated. It will play an important role in reducing the calculation time when users require positioning over a large area, and where the fingerprint database is huge.

## 4. Novel Fusing Positioning Algorithm

### 4.1. Algorithm Framework for Indoor Continuous Positioning on the Move

**Figure 4:**
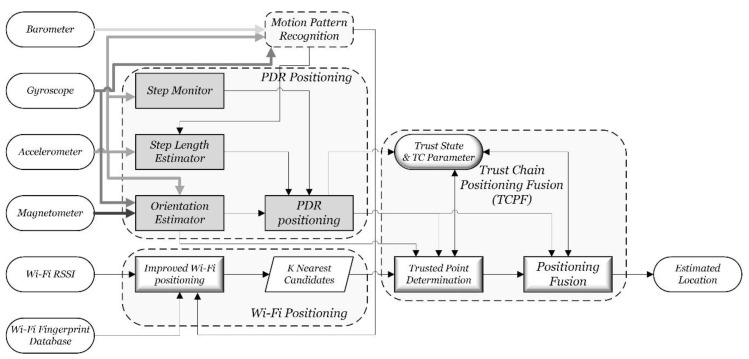
Algorithm framework of indoor continuous positioning.

The overall algorithm can be divided into four modules: the motion pattern recognition module, the Wi-Fi positioning module, the PDR positioning module, and the Trust Chain Positioning Fusion module. They are shown as dashed rectangles with rounded edges in Figure 4. The inputs of the algorithm include acceleration, magnetic force, angular acceleration, pressure and Wi-Fi signals, which all can be obtained real time by a smartphone. The Wi-Fi fingerprint database is built up during the offline phase.

The motion pattern recognition module fuses the sensor data from the acceleration, gyroscope and barometric to identify the motion pattern of users in order to adopt different positioning algorithms. The Wi-Fi positioning module uses the improved Wi-Fi positioning algorithm described in Section 3 to get K most possible positioning estimations. The PDR positioning module fuses the sensor data from the acceleration, gyroscopes and magnetometers to realize indoor users’ displacement estimation. The *TCPF* module includes three parts, trusted point determination, trust state and positioning fusion algorithm. It determines the trusted points by matching Wi-Fi positioning results with the annular sector set up by PDR positioning. A finite state machine is used to establish the dynamic states of the continuous positioning locking mechanism. Finally, Wi-Fi and PDR positioning results are fused based on a dynamic positioning fusion algorithm to get the best positioning estimation.

Multi-sensor data can be used to recognize motion patterns, such as walking with hand-held smartphones, walking with hand swinging, static standing, and so on; in total, six of the most common motion patterns. The recognition accuracy can reach 95% [30]. The upstairs and downstairs motion can also be identified with the assistance of a barometer [17]. For simplicity, the details of this part are omitted. The Wi-Fi positioning module is explained as Section 3, and the following focuses on the other two modules of the algorithm.

### 4.2. PDR Positioning Algorithm

The PDR positioning module monitors the walking action of the user, then estimates their step length and orientation to estimate the displacement so that positioning estimation can be realized, expressed as Equation (7).

(7) $$L_{t|t-1}=L_{t-1|t-1}+L_{△PDR}^{\text{t}|t-1}\text{=}L_{t-1|t-1}+l_{\text{t}}\left(\begin{array}{c} \cos\theta_{t}\\ \sin\theta_{t} \end{array}\right)$$

where $L_{t-1|t-1}$ is the latest positioning result ${\left(X_{t-1},Y_{t-1}\right)}^{T}$ at epoch t, $L_{t|t-1}$ is the PDR positioning result, $l_{t}$ is the estimated step length, and $\theta_{t}$ is the estimated orientation.

It is important to note that the main researches are based on the hypothesis that smartphones keep a specific attitude in the positioning process. Main attitudes under consideration include hand-held [11,31], kept in a pocket [31], or tied on the waist [32], and so on. The real-time coordinate transformation of the built-in sensor data is an extremely complex process, even without a solution, because the position and the attitude of smartphones are random and vary over time. It is assumed that smartphones maintain a hand-held attitude during the whole process of positioning, while it is assumed in this paper that users keep watching the phone at the same time. It is the most common attitude when smartphones are used for navigation, and the orientation of the smartphone (y axis) stays the same with the user’s.

The PDR positioning algorithm can be divided into three parts: the step monitoring, the step length estimation, and the orientation estimation. The step monitoring algorithm uses the rising edge of the average acceleration, assisted by the adjacent step time difference limitation to reduce misjudgment [11], and to identify every step. The step length estimation algorithm under walking conditions is shown in Equation (8).

(8) $$l_{\text{t}}=K_{w}{\left(a_{v,max}-a_{v,min}\right)}^{\frac{1}{4}}$$

where $l_{t}$ is the step length, $a_{v,max}-a_{v,min}$ is the peak-to-peak value of vertical acceleration $a_{v}$ during each step, $K_{w}$ is a coefficient calibrated for individuals. The step length is a personalized parameter [31], and has correlation with user’s height, leg length, weight, and habits. Higher accurate step length estimation needs further personalized correction fusing user’s historical trajectory [11].

The orientation estimation algorithm fuses sensor data from an electronic compass and a gyroscope by Kalman filter to get the best estimation [31,32]. The estimation algorithm is based on the hand-held attitude, a specific optimization algorithm is needed in other attitudes [33]. The angle, angular velocity and angular acceleration at epoch t is labeled as $Q_{t},V_{t}$, and $u_{t}$, respectively. The orientation variable is defined as $S_{t}={\left[Q_{t},V_{t}\right]}^{T}$. According to the Newton’s theorem, the system transfer equation is shown in Equation (9).

(9) $${\text{S}}_{\text{t}}=AS_{t-1}+Bu_{t}+w$$

where $A=\left[\begin{array}{cc} 1 & dt\\ 0 & 1 \end{array}\right]$ and B=[dt22dt] are coefficients, and w denotes the Gaussian noise of the system with zero mean and variance ϕ. The observation of the system comes from the azimuth reading of the compass, which is defined as $O_{t}$. The observation function can be expressed as Equation (10).

(10) $$O_{t}=CS_{t}+r$$

where C=[1 0] and r denotes the Gaussian noise of the magnetometer output with zero mean and variance φ. The fusion equation based on Kalman filter is expressed as Equations (11) and (12).

Predicting

(11) $$\begin{array}{l} {\text{S}}_{t|t-1}=AS_{t-1|t-1}+Bu_{t}\\ P_{t|t-1}=AP_{t-1|t-1}A^{T}+\phi \end{array}$$

Updating

(12) $$\begin{array}{l} {\text{K}}_{\text{t}}=P_{t|t-1}C^{T}{\left(CP_{t|t-1}C^{T}+\varphi \right)}^{-1}\\ S_{t|t}=S_{t|t-1}+K_{t}\left(O_{t}-CS_{t|t-1}\right)\\ P_{t|t}=\left(I-K_{t}C\right)P_{t|t-1} \end{array}$$

**PDR Positioning Error Analysis**

**Figure 5:**
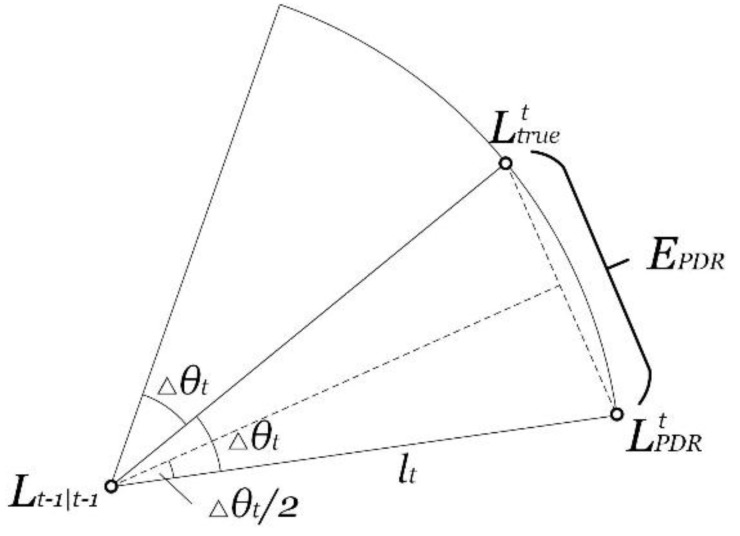
PDR positioning error estimation.

The PDR positioning results considering errors can be expressed as Equation (13).

(13) $$L_{t|t-1}=L_{t-1|t-1}+\left(l_{t}+△l_{t}\right)\left(\begin{array}{c} \cos\left(\theta_{t}+\Delta \theta_{t}\right)\\ \sin\left(\theta_{t}+\Delta \theta_{t}\right) \end{array}\right)$$

where the main source of the PDR positioning error is the step length estimation error $△l_{t}$ and the orientation estimation error $\Delta \theta_{t}$. When the user is normally walking indoors, the step length can be regarded as a constant so that $△l_{t}$ can be regarded as a constant much less than $l_{t}$. The PDR positioning error has a continuity property within a short distance, because $\Delta \theta_{t}$ is satisfied with a Gaussian distribution from the perspective of the statistics, which will be validated in Section 5.1, and the orientation variation is continuous and gradual. The PDR positioning error estimation is shown in Figure 5, and the mathematical expectation ${\text{E}}_{\text{PDR}}$ is shown in Equation (14).

(14) $${\text{E}}_{\text{PDR}}\text{=}2\int_{0}^{\frac{\pi }{2}}\frac{1}{\sqrt{2\pi \sigma^{2}}}\cdot {\text{e}}^{-\left(\frac{\Delta \theta_{t}^{2}}{2\sigma^{2}}\right)}\cdot 2l_{t}\cdot \sin\left(\frac{\Delta \theta_{t}}{2}\right)d\Delta \theta_{t}$$

### 4.3. Trust Chain Positioning Fusion Algorithm

#### 4.3.1. Algorithm Flow

**Figure 6:**
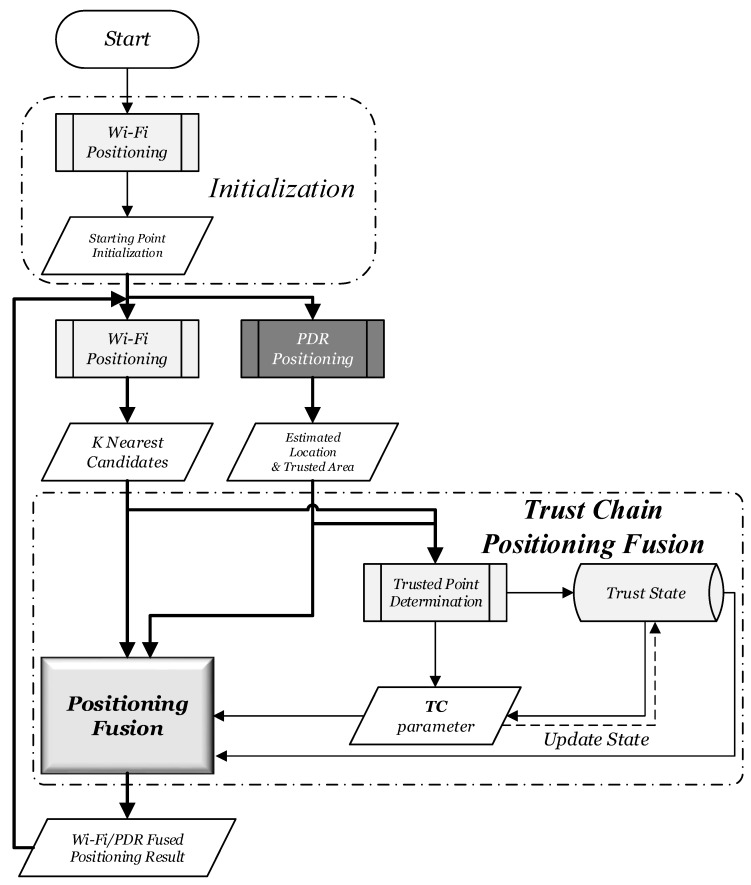
Algorithm flow of proposed fusing positioning.

The positioning fusion algorithm is the key to take respective advantage of the Wi-Fi positioning and the PDR positioning. The algorithm flow of the proposed fusion algorithm, which is named *Trust Chain Positioning Fusion* (*TCPF*) algorithm, is shown in Figure 6. The *TCPF* algorithm includes three parts, trusted point determination, trust state, and positioning fusion. The first step is to determine the trusted point by matching the Wi-Fi positioning result with the annular sector set up by the PDR positioning. The next step is to update the trust state defined by a finite state machine and the TC parameter based on the trust determination results. The final step is to fuse two positioning results based on the trust state and the TC parameter. Compared to the traditional Kalman Filter that directly fuses two positioning results, the proposed algorithm realizes the “process-level” fusion.

#### 4.3.2. Trusted Point Determination

Trusted point determination is the method to recognize the accurate positioning results, and it is the key to set up the trust chain. The key to improve the PDR/Wi-Fi fusion positioning accuracy is to periodically eliminate the cumulative error. However, estimating positioning error at each point is really difficult. The innovative idea is identifying trusted points instead. The key idea behind the *TCPF* algorithm is using the accurate point to support the whole positioning process in order to improve the positioning performance.

**Figure 7:**
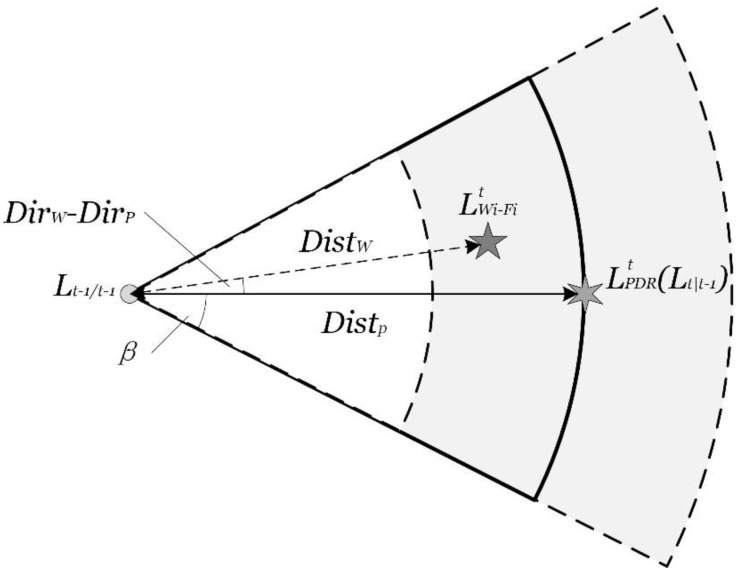
The trusted area set up by PDR positioning.

The trusted point is determined if the Wi-Fi positioning result falls within the annular sector set up by the PDR positioning. The annular sector is named the trusted area. It is based on the high accuracy of the PDR positioning over a a short distance. If the two positioning results meet the conditions in Equation (15), the real location located in the trusted area with high probability and accuracy. The trusted area is shown in Figure 7 with its mathematic expression as Equation (15).

(15) $$\left\{ \begin{array}{l} \left|Dist_{p}-Dist_{w}\right|\leq \rho \\ \left|Dir_{p}-Dir_{w}\right|\leq \beta \end{array}\right.$$

where $Dist_{w}$ and ${\text{Dir}}_{\text{w}}$ is respectively the distance and angle between the Wi-Fi positioning results and the latest positioning results. $Dist_{p}$ and ${\text{Dir}}_{p}$ are referred to as the PDR positioning. β is the tolerance parameter of the orientation estimation error, set as the experimental value 45°, and ρ is the tolerance parameter of the step length estimation error, set as the experimental value 2 m.

#### 4.3.3. Trust State

**Figure 8:**
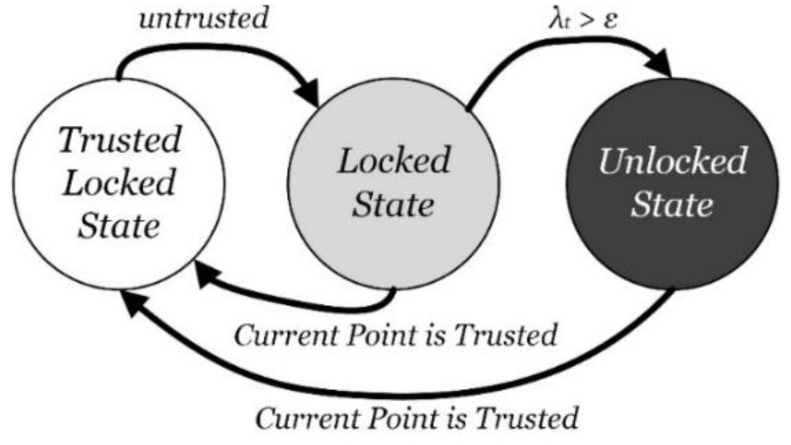
Finite state machine of trust state.

Trust state is established to mark the state of the trust chain, and is realized by a finite state machine shown as Figure 8. Three states are defined: *Trusted Locked State*, *Locked State* and *Unlocked State*. The Trusted Locked State is set when current point is determined as the trusted point. At this time, the positioning result is regarded as highly accurate, and the trust chain is locked. Until the positioning point is no longer consider the trusted point, the state changes to the Locked State. At this time, the trust chain locks the current point with the latest trusted point so that the positioning still mainly relies on the PDR positioning result due to its high accuracy over a short range. The Wi-Fi positioning candidate points are filtered by the trust area. If the point remains untrusted to a certain extent, the state would change to the Unlocked State. At this time, the Wi-Fi positioning is mainly relied on, and its candidate points are not filtered. As long as the current positioning point is determined as the trusted point, the state is change back to the Trusted Locked State, forming a full loop.

Where $\lambda_{t}$ is the *TC* parameter, which is introduced and analyzed in the next section, and ε is the determination factor to change the state from the Locked State to the Unlocked State. The parameter ε characterizes the locking extent from the latest trusted point to the current positioning point. The larger ε is, the more trust is given to the PDR positioning based on the trusted point, and vice versa. In the paper, the empirical value 1 is selected so that two positioning algorithms is balanced equivalently.

#### 4.3.4. Positioning Fusion

The positioning fusion estimation is the dynamic weighted sum of the PDR positioning estimation and the improved Wi-Fi positioning estimation, as shown in Equation (16). The PDR positioning is based on the built-in sensors data and the Wi-Fi positioning module is based on the RSSI of the Wi-Fi. They are independent of each other so that they can be regarded as the unrelated positioning estimations for the linear weighted fusion.

(16) $$L_{\text{t|t}}=\frac{L_{t|t-1}+\lambda_{t|\text{t}-1}L_{Wi-Fi}^{t}}{1+\lambda_{t|t-1}}$$

where $L_{\text{t|t-1}}$ and $L_{Wi-Fi}^{t}$ is respectively the PDR and Wi-Fi positioning result at epoch t, and $L_{\text{t|t}}$ is the positioning fusion result ${\left({\text{X}}_{t},Y_{t}\right)}^{\text{T}}$ at epoch t. $\lambda_{t|t-1}$ is the key weighting parameter, which is named the *TC* parameter.

The first core issue of the algorithm is the *TC* parameter, which is used to characterize the inaccuracy (or mistrust) of the positioning point. On one hand, it is defined as increasing linearly as the positioning target moves according to the continuity property of the PDR positioning error, as Equation (17).

(17) $$\lambda_{t|t-1}=\lambda_{t-1|t-1}+d_{PDR}\cdot \alpha$$

where $d_{PDR}$ is the distance target moves during epoch t−1 an t, and α is a constant coefficient, which is the ratio of the accumulated error of the PDR positioning moving one meter and the Wi-Fi average positioning error. The Wi-Fi positioning error is regarded as a Gaussian random error so that the average is set as the metric of the inaccuracy. On the other hand, the *TC* parameter is updated after fusing the Wi-Fi positioning result. When the positioning point is no longer considered as the trusted point, the *TC* parameter is iterated after fusion, as Equation (18). The inaccuracy of the fused result is calculated as the $\left(1+\lambda_{t|t-1}\right)$ times the original accumulated inaccuracy $\lambda_{t|t-1}$.

(18) $$\lambda_{t|t}=\lambda_{t|t-1}(1+\lambda_{t|t-1})$$

Otherwise, the trust state is changed back to Trusted Locked State, and the TC parameter is changed to zero.

The second core issue of the algorithm is the relationship between the trust state and the Wi-Fi positioning. The Wi-Fi positioning result $L_{Wi-Fi}^{t}$ is defined as follows.

1. At the locked state, including Trusted Locked State and Locked State, the Wi-Fi positioning candidate points are limited within the trust area, and $L_{Wi-Fi}^{t}$ is the weighted sum of these selected points’ locations.
2. At the Unlocked State, the Wi-Fi positioning is not filtered, and the candidate points are all weighted added as the positioning fusion estimation $L_{Wi-Fi}^{t}$.

Taking overall Equation (7) and Equations (16)−(18) into account, the proposed *TCPF* algorithm is expressed as Equation (19).

(19) $$\begin{array}{c} L_{t|t-1}=L_{t-1|t-1}+L_{\Delta PDR}^{\text{t|t-1}}\\ \lambda_{t|t-1}=\lambda_{t-1|t-1}+d_{PDR}\cdot \alpha \\ L_{\text{t|t}}\text{=}L_{\text{t|t-1}}+\frac{\lambda_{t|t-1}}{1+\lambda_{t|t-1}}\left(L_{W\text{i-Fi}}^{t}-L_{t|t-1}\right)=\frac{L_{\text{t|t-1}}+\lambda_{t|t-1}L_{W\text{i-Fi}}^{t}}{1+\lambda_{t|t-1}}\\ \begin{array}{c} \lambda_{t|t}=0,\;if\;Trusted\;Locked\;State\;\\ \lambda_{t|t}=\lambda_{t|t-1}(1+\lambda_{t|t-1}),\;otherwise \end{array} \end{array}$$

Compared to the traditional fusion algorithm, the proposed *TCPF* algorithm has the following two significant features.

1. The trusted point determination method and the trust state machine are established to multi-dimensionally adjust the weight of the dynamic positioning fusion so that the fused result is the optimal estimation.
2. The algorithm has a strong anti-interference performance without error cycles. At the locked state, the *TC* parameter is set as a small value so that the fused positioning result is focused on the PDR positioning, which is highly accurate over a short distance. At the unlocked state, the *TC* parameter increases rapidly iteratively so that the fused positioning result is focused on the Wi-Fi positioning. The weight parameter is dynamically adjusted so that there is no error cycle.

## 5. Experimental Evaluation

### 5.1. Experimental Setup

The continuous positioning on the move experiment (called Experiment 2) is set up to evaluate the performance of the proposed fusing positioning algorithm, including the improved Wi-Fi positioning, the PDR positioning, and the novel *TCPF* algorithm.

**Figure 9:**
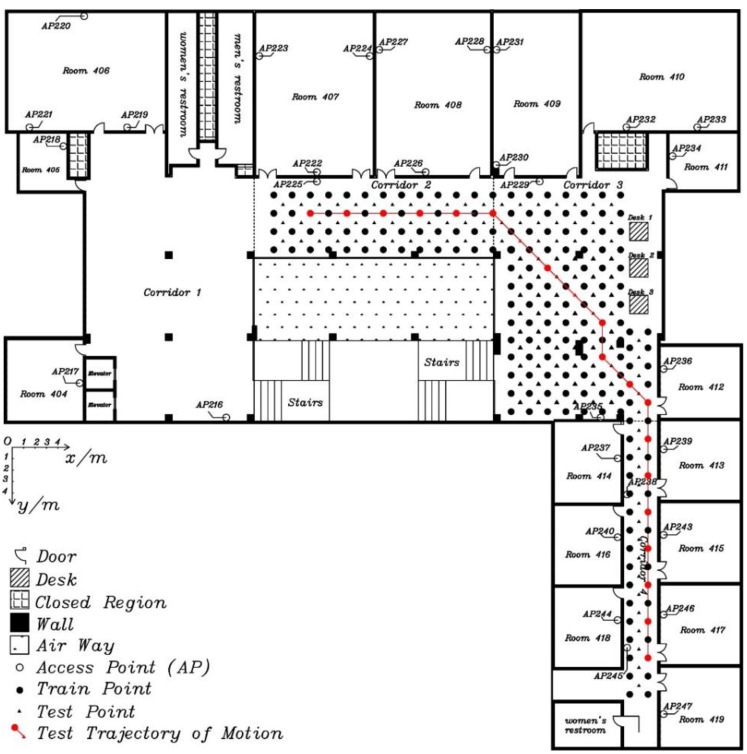
The layout of the experimental area.

In order to sufficiently test positioning performance, including turning on the move, the experimental field is changed to the 4th floor in the LiJiao Building, Peking University, which is a typical modern teaching building with a larger area than where Experiment 1 was performed. The layout of the experiment area is shown in Figure 9. Altogether, 30 APs, marked as hollow dots, are evenly distributed in the experimental area, which is about 430 m^2^. Altogether, 184 fingerprint points, marked as black and red solid dots, are evenly distributed and the adjacent interval, aligned at the floor tile, is 1.6 m. The moving path for testing, marked as a red line and red points, includes 20 test points with a length of 60.6 m.

**Figure 10:**
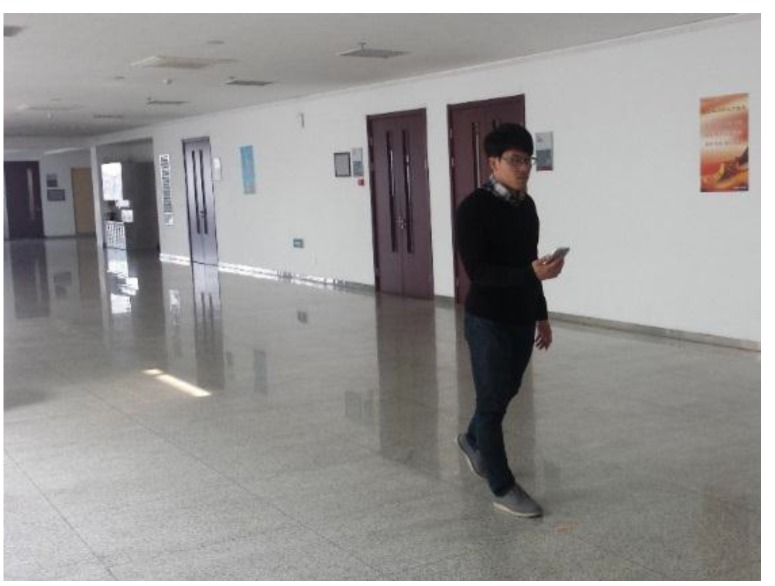
Attitude of the smartphone and the user’s gesture in the experiment.

The Experiment 2 is divided into two parts: the static positioning part and the continuous positioning on the move part. In the former part, the static test data is sampled 10 times at each test point, and the sampling interval is 0.5 s. In the latter part, the tester walks at a constant speed along the moving path, and starts Wi-Fi positioning once passing a test point. The built-in sensor data is automatically recorded in the background during the entire moving process, and the sampling interval is set at 50 ms. The moving test is repeated 10 times, and the orientation of the smartphone conforms with the user’s all the time. The attitude of the smartphone and the tester’s gesture are shown in Figure 10. The train data is the same for the two parts, and is sampled 10 times at each fingerprint point with 0.5 s intervals.

The following analysis is divided into three parts, including the improved Wi-Fi positioning evaluation, the PDR positioning evaluation and the fusing positioning evaluation.

### 5.2. Improved Wi-Fi Positioning Evaluation

**Figure 11:**
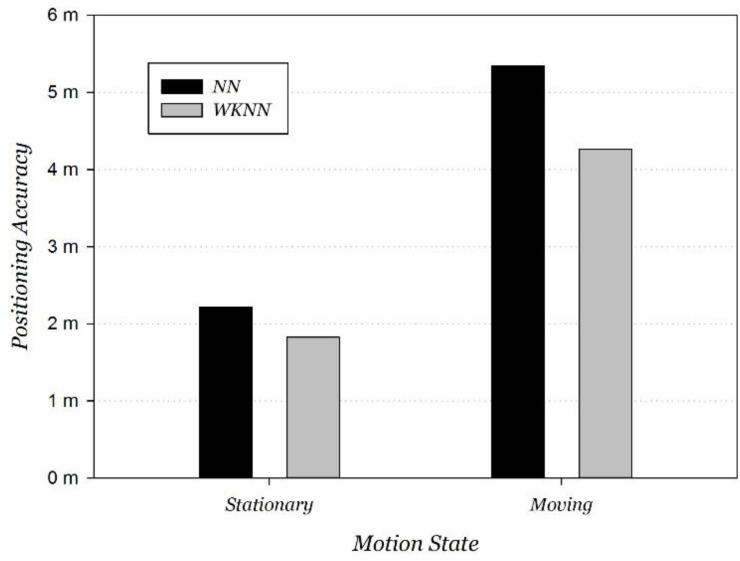
Wi-Fi positioning performance at moving/stationary states.

Firstly, a positioning performance comparison at stationary and moving states was carried out. The NN and WKNN positioning algorithms were adopted. Compared to the positioning error at stationary state, error on the move increased by 141% and 133%, respectively, using NN and WKNN positioning algorithms. The average positioning error at moving state respectively deteriorated to 5.35 m and 4.27 m, and the comparison is shown in Figure 11.

**Figure 12:**
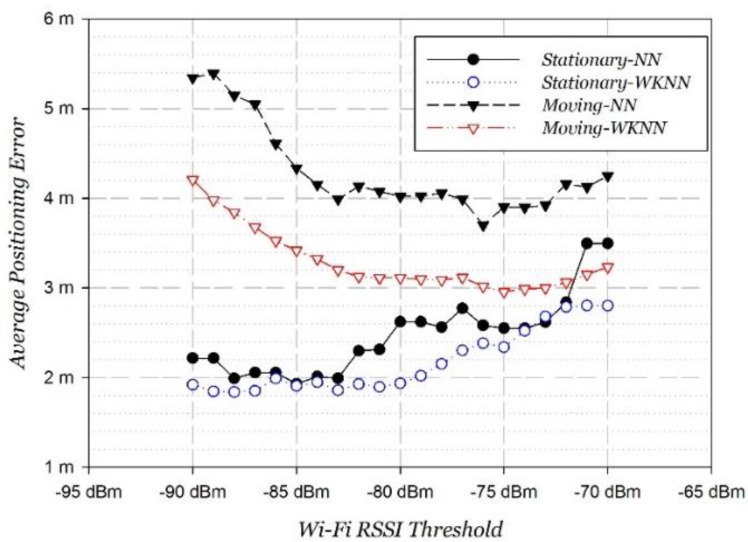
Wi-Fi positioning performance with RSSI threshold.

Secondly, we adjusted the RSSI threshold step by step to find the variation tendency of the Wi-Fi positioning error at stationary and moving states, as shown in Figure 12.

Two conclusions can be summarized from the tendency of the positioning errors.

1. For Wi-Fi stationary positioning, weak signals less than −75 dBm also contributed to positioning so that the excessive threshold reduced signal information and increased the positioning error. Overall, Wi-Fi static positioning achieved a strongest performance at around −85 dBm.
2. For Wi-Fi moving positioning, the positioning error firstly decreased as the threshold increased, which was between −90 and −75 dBm. Then, as the threshold increased continuously, the positioning error increases instead. It can be explained that weak signals less than −75 dBm cause a decline in performance due to the great instability on the move. The best performance is obtained at around −75 dBm. This is in accordance with the properties of Wi-Fi signals on the move analyzed in Section 3.1.

**Figure 13:**
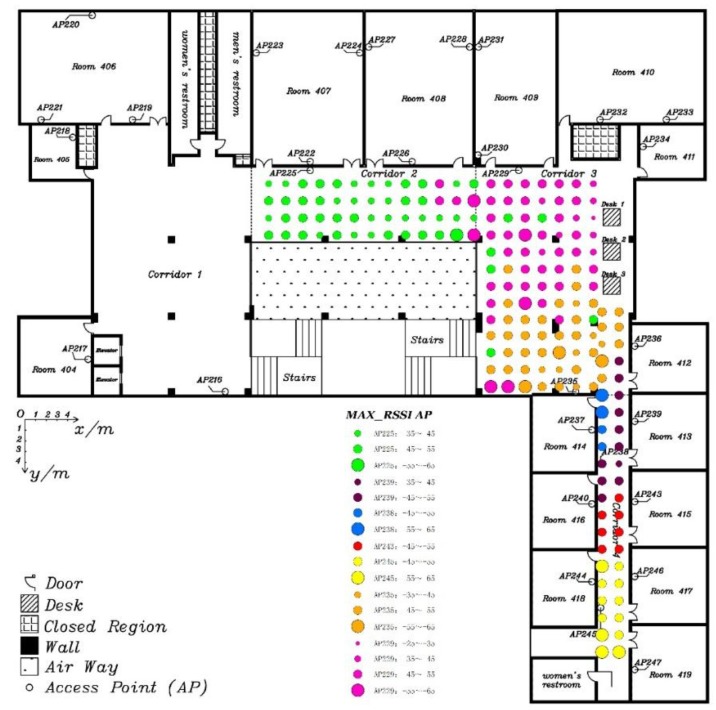
Strongest AP distribution in experimental area.

Next, the clustering property of the strongest AP was validated through the strongest AP distribution in the experimental area, as shown in Figure 13. We found that the distribution has an extremely strong clustering property.

**Figure 14:**
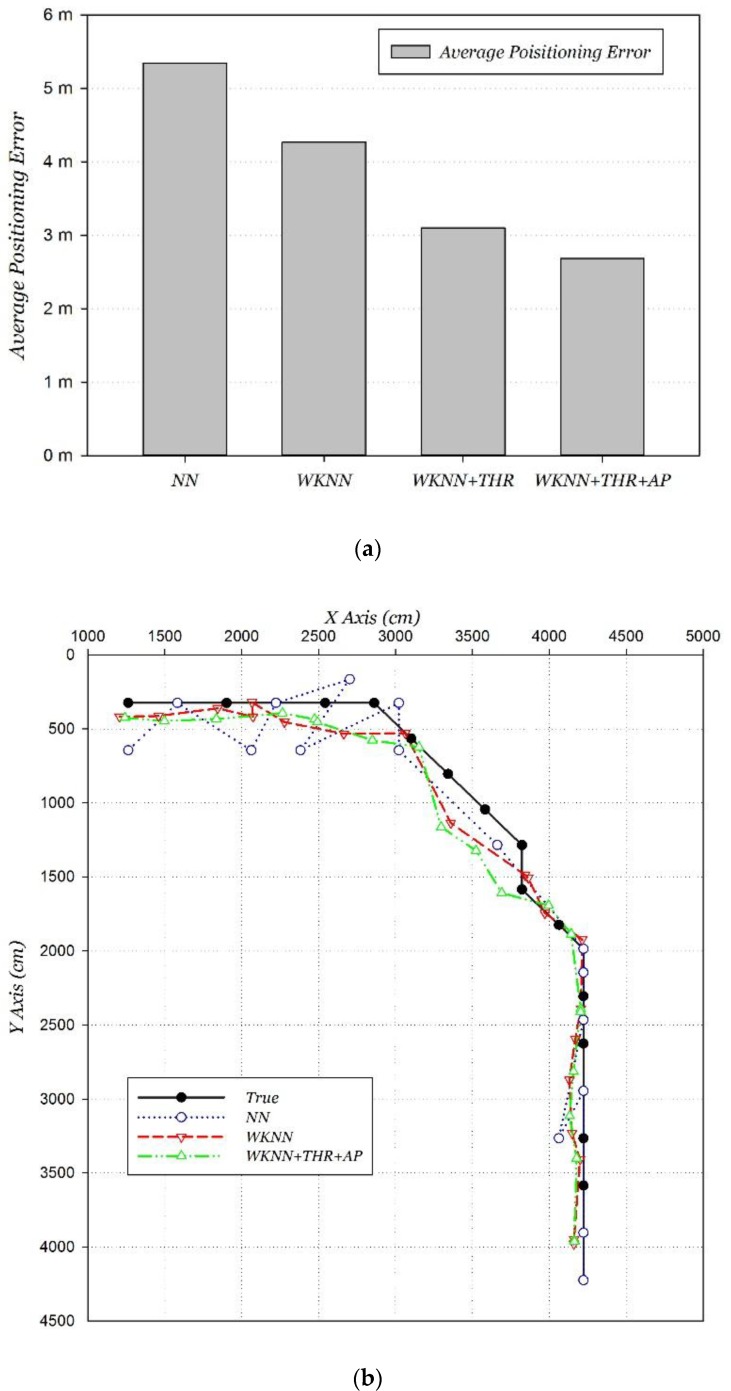
Performance of improved Wi-Fi positioning. (**a**) Accuracy; (**b**) distribution.

Finally, the improvement to the classic positioning algorithm by the two proposed methods is analyzed step by step, and the positioning accuracies and distributions that were compared are shown in Figure 14. The “WKNN + THR” algorithm represents the classic WKNN algorithm fusing with the dynamically adjusting RSSI threshold method, and it fuses the AP matching method representing the “WKNN + THR + AP” algorithm. The experimental results show that the proposed two methods have a significant effect in improving the Wi-Fi positioning performance on the move. The average error of the improved Wi-Fi positioning was reduced to 2.68 m.

### 5.3. PDR Positioning Evaluation

**Figure 15:**
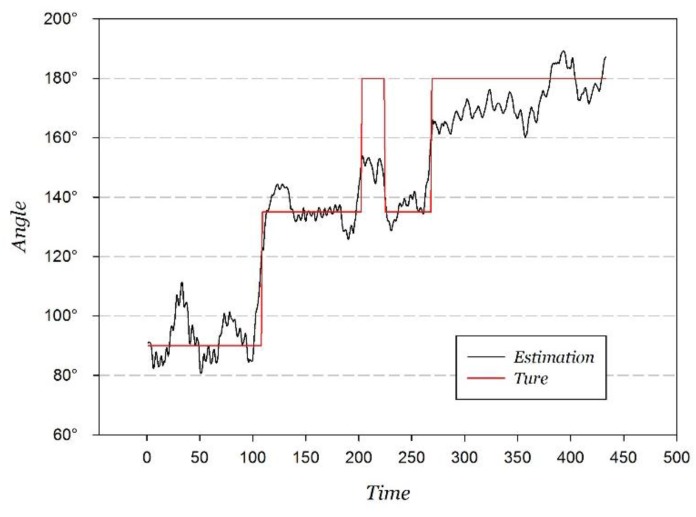
Orientation estimation and true value.

As the key part of the PDR positioning, the orientation estimation was firstly analyzed, and the comparison between the orientation estimation and the true value at the hand-held attitude is shown in Figure 15.

**Figure 16:**
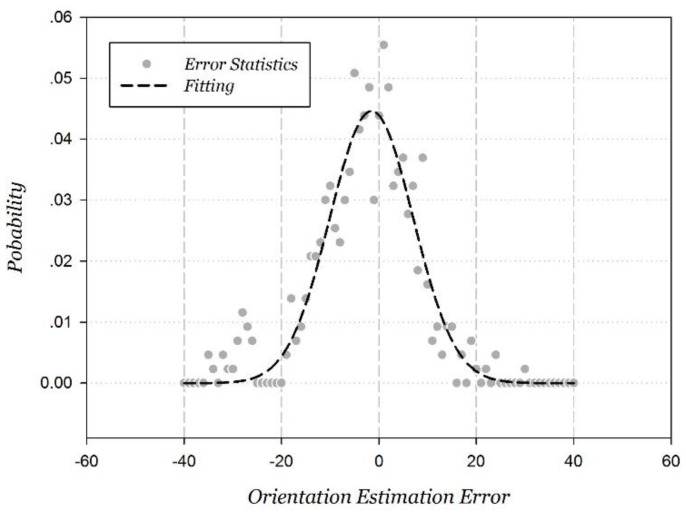
Gaussian fitting orientation measurement error.

Through the statistical analysis of the orientation estimation error, the distribution approaches a Gaussian distribution, and the fitted result is shown in Figure 16.

The Gaussian fitting correlation coefficient is 0.9520, and the fitted estimation error probability distribution function ${\text{e}}_{PDR}$ is shown in Equation (20).

(20) $${\text{e}}_{PDR}=\frac{1}{\sqrt{2\pi \sigma^{2}}}e^{-\frac{{\left(\theta -\theta_{0}\right)}^{2}}{2\sigma^{2}}}$$

where $\sigma =8.5916,\;x_{0}=-1.5086$. The statistical probability is 94.00% when the orientation estimation error is within ±20°, 87.76% corresponding to within ±15°, and 71.13% corresponding to within ±10°.

Based on the fitted Gaussian distribution and Equation (14), the mathematical expectation of the PDR positioning error is 8.9675 cm when the step length estimation d is estimated as 75 cm. It is the important coefficient used to calculate the parameter α in Equation (17).

**Figure 17:**
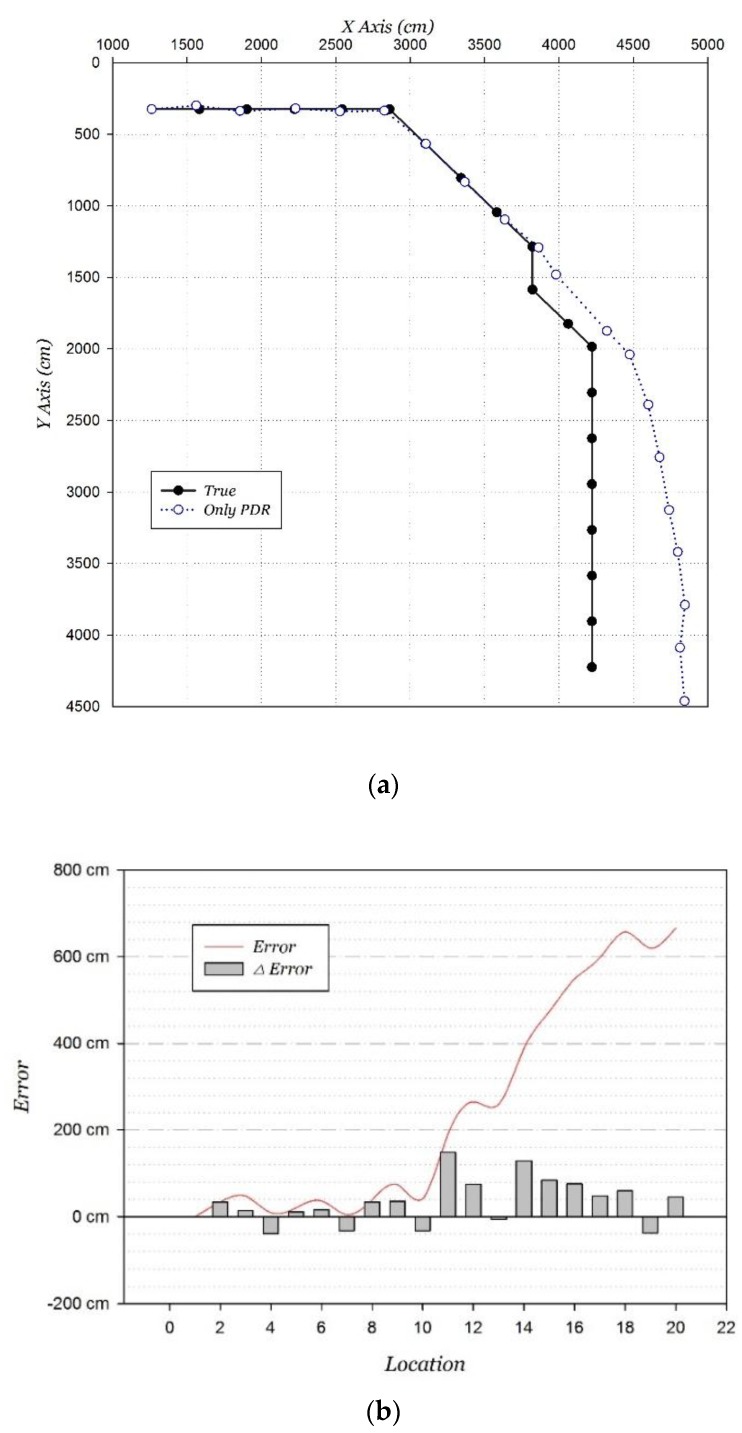
PDR positioning. (**a**) Distribution; (**b**) error distribution.

In order to evaluate the performance of the only PDR positioning, the initial location is set as the starting point. The positioning results and accumulated errors are shown in Figure 17. The accumulated error reaches 6.66 m. The experimental results show that the PDR positioning is extremely accurate over a short distance in the linear movement. Positioning errors mainly appear in turnings, because of the orientation estimation error. Overall, the PDR positioning error varies almost linearly over a short distance.

### 5.4. Fusing Positioning Evaluation

**Figure 18:**
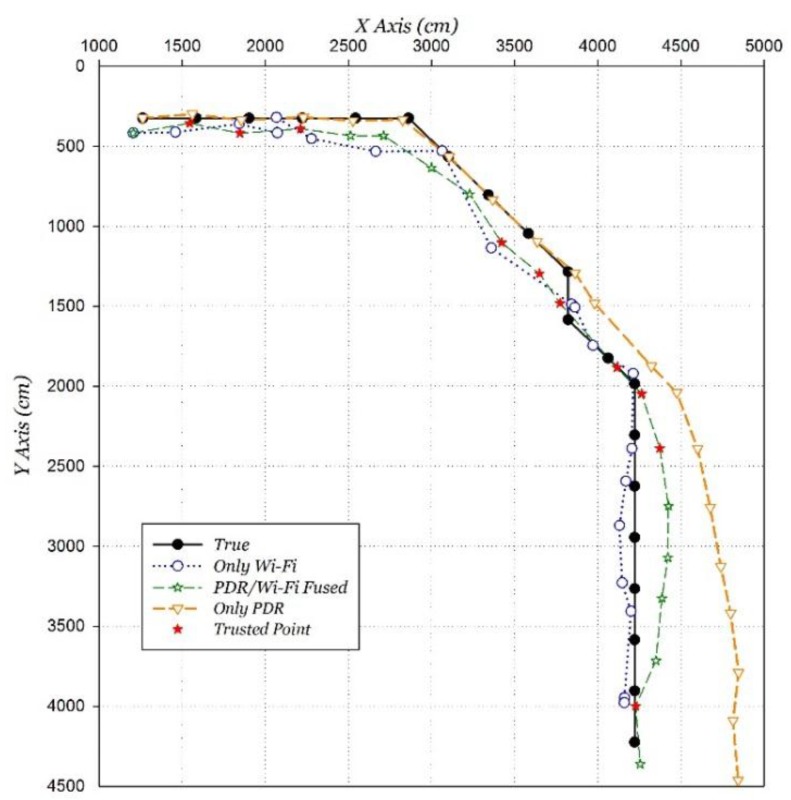
PDR/Wi-Fi fusion positioning result distribution.

The PDR/Wi-Fi fusing positioning based on the proposed algorithm is compared with the only Wi-Fi positioning and the only PDR positioning, as shown in Figure 18. The result shows that the fusion positioning performance is much better than the only Wi-Fi or PDR positioning. The average positioning error of the proposed algorithm is 1.36 m, a decrease of 49.6% compared to that of the only improved Wi-Fi positioning.

The proposed algorithm demonstrates strong anti-interference performance verified by the behavior of correcting the PDR accumulated error in the second half of the path. The proposed algorithm can dynamically adjust the weight, and take advantage of two positioning algorithms to obtain a better positioning performance. The point with a red painted marker “★” is the trusted point, and its average positioning error is 1.10 m. It plays a role as the “anchor” throughout the positioning process due to its higher positioning accuracy than other positioning points.

**Figure 19:**
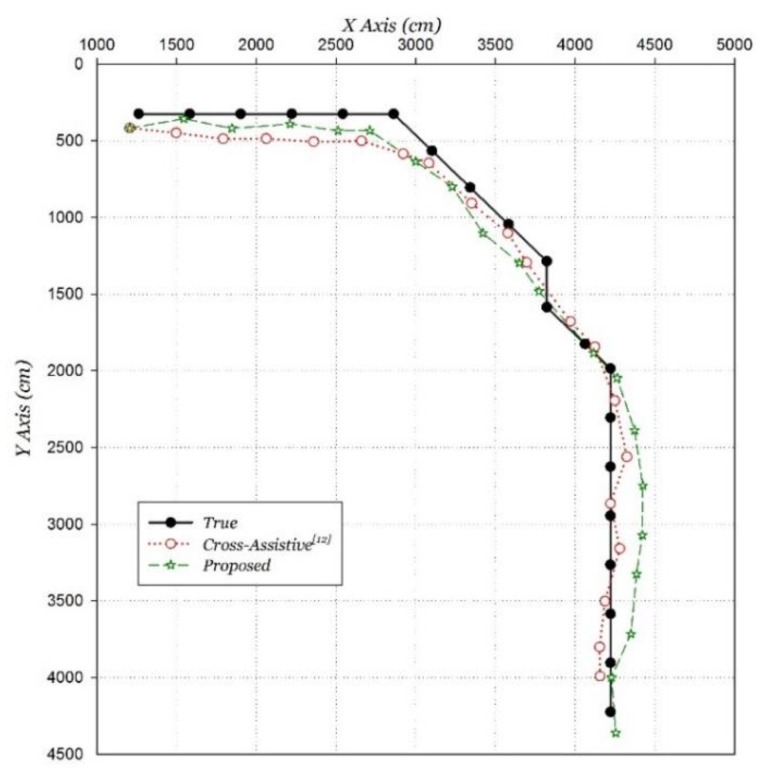
Lateral comparison of proposed algorithm.

The proposed algorithm was compared to the similar fusing positioning algorithm, which is the Cross-Assistive algorithm [12]. The positioning comparison results are shown in Figure 19. The average positioning error is 1.91 m using the Cross-Assistive algorithm with the same improved Wi-Fi positioning algorithm, with 28.8% less accuracy than the proposed algorithm.

## 6. Conclusions and Future Work

This paper has proposed a novel indoor continuous positioning algorithm fusing built-in sensors and Wi-Fi on smartphones to deal with positioning problems on the move, such as large errors and poor stability, which cannot be well dealt with in traditional algorithms. Compared to the traditional algorithms, the main innovative points include an improved Wi-Fi positioning algorithm and a novel positioning fusion algorithm called the *TCPF* algorithm. The improved Wi-Fi positioning algorithm enhances the Wi-Fi positioning performance on the move by dynamically adjusting the RSSI threshold and AP matching. It is based on the properties of Wi-Fi signals at moving state, which are analyzed through a novel “quasi-dynamic” Wi-Fi signal experiment. The *TCPF* algorithm is proposed to realize the “process-level” fusion of Wi-Fi and PDR positioning. The algorithm is described in detail in three parts: Trusted point determination, trust state, and positioning fusion. The mathematical calculation model is also summarized and verified by a field experiment, which was carried out in a typical teaching building. The result shows that the average positioning error on the move using the improved Wi-Fi positioning algorithm decreases by 49.8% compared to the traditional algorithm. The average positioning error of the novel fusing positioning algorithm reaches 1.36 m, a decrease of 28.8% compared to a current fusion algorithm. The proposed algorithm can effectively reduce the influence caused by the unstable Wi-Fi signals, eliminating the positioning error accumulated over time, and improving the accuracy and stability of indoor continuous positioning on the move.

There is still room for improvement in the algorithm. The main problem is that the algorithm greatly relies on the smartphone’s attitude and the user’s gesture. Although the attitude under consideration is among the most common one, the smartphone and user are flexible and their behabiours vary in daily life. How to accurately recognize the various attitudes and adopt corresponding compensation algorithms is the key to further increasing the universality of the algorithm’s application. Other research directions include hardware differentiation, map matching, and power control. The adaptation problem for different smartphones, especially for the varying accuracies of built-in sensors, need to be further studied. The map matching technology application has been mainly used in outdoor scenarios, but lacks application in indoor environmenst. Further research could focus on motion recognition with indoor spatial analysis.
